# An Accessory Extensor Digitorum Longus Muscle – a Rare Variation

**DOI:** 10.15388/Amed.2024.31.2.2

**Published:** 2024-12-04

**Authors:** Saphal Lakshmi Pasupulati, Sanjit Satheesan, Piyush Saiyam, Saketh Chowdary, P K Sankaran, Kishore Sesham

**Affiliations:** 1 All India Institute of Medical Sciences, Mangalagiri, India; 2 Department of Anatomy, All India Institute of Medical Sciences, Mangalagiri, India

**Keywords:** lower limb, anatomic variation, skeletal muscle, tendons, leg, extensor digitorum longus, accessory extensor digitorum longus, Raktažodžiai: apatinė galūnė, anatominiai pokyčiai, skeleto raumenys, sausgyslės, koja

## Abstract

During the routine dissection of lower limb in a female cadaver of age 61 years, a rare variation in the extensor digitorum longus (EDL) muscle was noticed on the both legs. An EDL muscle with small belly dividing into two slips, which inserted on to the second and third digits on the right leg, whereas on the left leg divided into three slips, which inserted on to the second, third and fourth digits. There was also an accessory EDL muscle arising from the middle third of the medial surface of the fibula and inserted on to the fifth digit in the left foot, and it inserted on to the fourth and fifth digits in the right foot. To our knowledge, such variation hasn’t been reported in literature and apprehension of such variations is important for surgeons and orthopaedicians with regards to contractures and surgical procedures concerning the anterior leg and dorsum of the foot.

## Abstract (it)

Atliekant įprastinį 61 metų moteriškos lyties lavono apatinės galūnės skrodimą, pastebėta reta ilgojo tiesiamojo raumens (angl. *extensor digitorum longus*, EDL) variacija abiejose kojose. EDL raumuo su nedideliu pilvuku dalijosi į dvi skiltis, kurios įeina į dešinės kojos antrąjį ir trečiąjį pirštus, o kairės kojos raumuo dalijosi į tris skiltis, kurios įeina į antrąjį, trečiąjį ir ketvirtąjį pirštus. Taip pat buvo papildomas EDL raumuo, kylantis iš kairiosios kojos blauzdikaulio medialinio paviršiaus vidurinio trečdalio ir įsiterpiantis į kairės kojos penktąjį pirštą, o dešinės kojos – į ketvirtąjį ir penktąjį pirštus. Mūsų žiniomis, literatūroje apie tokius pokyčius nebuvo rašyta, o chirurgams ir ortopedams yra svarbu juos suvokti, atsižvelgiant į kontraktūras ir chirurgines procedūras, susijusias su priekine kojos dalimi ir pėdos nugarine dalimi.

## Introduction

The muscles of the anterior compartment of the leg consist of tibialis anterior, extensor hallucis longus, extensor digitorum longus (EDL) and the peroneus tertius (PT). The EDL is a unipennate muscle that arises from the anterior intermuscular septum of leg, the lateral condyle of the tibia, upper three-quarters of the medial surface of the fibula and adjacent interosseous membrane [1]. The tendon of EDL passes deep to the superior and inferior extensor retinaculum along with that of FT and gives rise to four tendinous slips forming the dorsal digital expansion with the tendon of extensor digitorum brevis inserting on to the dorsal surface of the middle and distal phalanges of the second, third, fourth, fifth digits. The PT arises from the distal third of the medial surface of the fibula, the anterior intermuscular septum and the adjacent interosseous membrane and inserts on to the medial aspect of the dorsal surface of the base of the fifth metatarsal. Both of their innervations are by the deep fibular nerve branch of the common fibular nerve, and they receive vascular supply from the anterior tibial artery and the fibular artery [4].

The primary function of the EDL is to extend the lateral four toes acting synergistically during dorsiflexion at the ankle joint [5]. The role of EDL in the gait cycle is prominent in dorsiflexing between preswing and initial contact [6] permitting the stance phase to be sturdy.

## Case Report

During the routine anatomical lower limb dissection in a female cadaver of age 61 years, unusual attachments of EDL were noticed in both lower limbs. The EDL muscle in this cadaver originated as two separate muscle bellies and it was named as EDL and accessory EDL.

**Figure. 1A:**
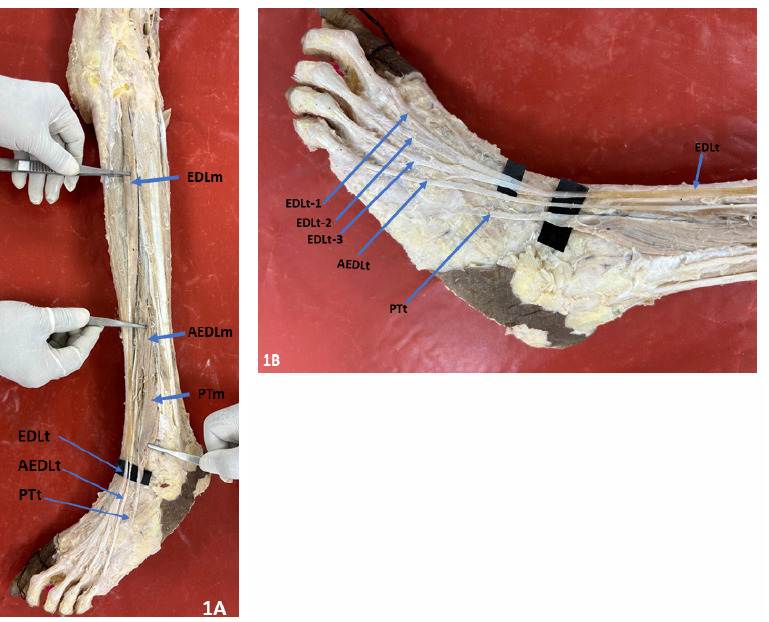
Left leg showing the small belly of EDL muscle and higher tendon formation, Accessory EDL muscle and PT muscle along with their tendons. **Figure. 1B:** Left foot showing the EDL tendon dividing into 3 slips, accessory EDL muscle tendon inserting into 5^th^ digit and FT inserting into base of fifth metatarsal. (Abbreviations: **EDLm** – Extensor digitorum longus muscle; **AEDLm**, Accessory Extensor digitorum longus muscle; AEDLT, Accessory Extensor digitorum longus tendon. **EDLt**, Extensor digitorum longus tendon; **EDLt-1**, 1^st^ tendon slip of Extensor digitorum longus; **EDLt-2**, 2^nd^ tendon slip of Accessory Extensor digitorum longus, **EDLt-3**, 3^rd^ tendon slip of Accessory Extensor digitorum longus **AEDL**, Accessory Extensor digitorum longus; **AEDLt-1**, 1^st^ tendon slip of Accessory Extensor digitorum longus; **AEDLt-2**, 2^nd^ tendon of Accessory Extensor digitorum longus; **PTt**, Peroneus tertius tendon.)

**Figure. 2A:**
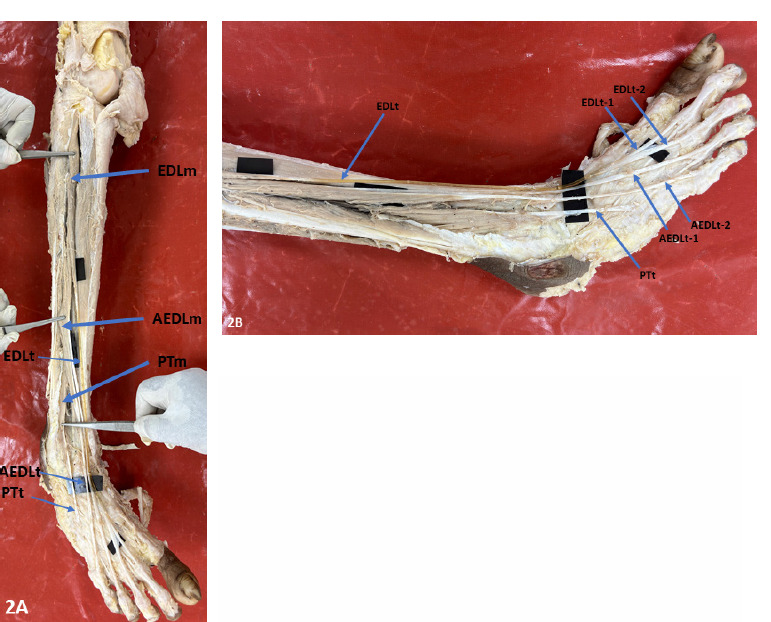
Right leg showing the thin EDL muscle belly and tendon, Accessory EDL muscle belly and tendon, Peroneus tertius muscle belly and tendon. **Figure 2B**. Right leg and dorsum of foot showing the course of the tendons of EDL dividing into 2 slips for 2^nd^ and 3^rd^ toes, Accessory EDL dividing into 2 slips for 4^th^ and 5^th^ toes (AEDLt-1 and AEDLt-2) and Peroneus tertius inserting into base of the 5^th^ metatarsal. (Abbreviations: **EDLm** – Extensor digitorum longus muscle; **AEDLm**, Accessory Extensor digitorum longus muscle; **AEDLT**, Accessory Extensor digitorum longus tendon. **EDLt**, Extensor digitorum longus tendon; **EDLt -1**, 1^st^ tendon slip of Extensor digitorum longus; **EDLt -2**, 2^nd^ tendon slip of Accessory Extensor digitorum longus, **AEDL**, Accessory Extensor digitorum longus; **AEDLt-1**, 1^st^ tendon slip of Accessory Extensor digitorum longus; **AEDLt-2**, 2^nd^ tendon of Accessory Extensor digitorum longus; **PTt**, Peroneus tertius tendon.)

The EDL muscle belly on both sides was peculiarly small, originated from the lateral condyle of tibia, anterior intermuscular septum, interosseous membrane and from the upper one-third of the medial surface of the fibula. It lacked its origins from the middle one-third of the medial surface of the fibula. Due to the EDL’s peculiarly small size, its tendinous formation occurred at the end of the upper one-third of the fibula, which is unusually high **(Figure 1 and 2)**.

In the left lower extremity, the tendon of EDL gave rise to three slips in the dorsum of foot which inserted on to the 2nd, 3rd, and 4th digits. The AEDL muscle originated from middle one-third of fibula, became tendinous in lower one-third of leg and inserted into 5th digit **(Figure 1)**.

In the right lower extremity, the EDL muscle belly gave a tendon which divided into two slips at the level of metatarsals and inserted on to the 2nd and 3rd digits through the dorsal digital expansion. The AEDL muscle originated from middle one-third of fibula above peroneus tertius origin and gave two tendinous slips in the lower one-third of leg only that are inserted into 4th and 5th digits **(Figure 2)**.

The EDL, PT and the accessory EDL gave rise to tendons which passed deep to the superior and inferior extensor retinaculum. The tendon of tibialis anterior, tendon of extensor hallucis longus, anterior tibial artery, deep fibular nerve, EDL, accessory EDL, PT were arranged deep to the extensor retinaculum from the medial side to the lateral side. In both the limbs, the PT originated from the distal third of the medial surface of the fibula, anterior intermuscular septum and inserted at the base of the 5th metatarsal. The tibialis anterior and extensor hallucis longus were normal.

## Discussion

There are several studies reported on variations in the EDL muscle in terms of its origin, insertion, number of tendinous slips and its communications to the adjacent muscles [2]. In this study there was a variation in EDL muscle belly and its tendinous divisions and there was an accessory EDL observed in the both legs. Kamasak et al. [4] detected the presence of two tendons from the EDL unilaterally, in which both the tendons divided into two slips and gave insertions equivalent to that of a usual EDL. The EDL is a unipennate muscle, but Wegiel et al. [5] reported a double headed EDL with upper belly giving a tendon inserting on to the phalanges of the 2nd and 3rd digits and lower belly tendon dividing into three slips and inserting into 3rd, 4th and 5th digits. Similarly, in this case there was a separate accessory muscle belly along with EDL dividing into tendons and inserting into specific toes.

Abhinitha et al. [6] noticed variation in the EDL tendon dividing into three slips and inserting into the phalanges of the second, third, fourth digits and gave a thin fibrous slip to FT. The tendon of FT was thicker than usual and bifurcated into slips which inserted on to the fifth digit’s phalanges and the base of fifth metatarsal. Sakuma et al. [7] noticed the absence of a fourth slip from the EDL being replaced by tendon of extensor digitorum brevis. These variations of the EDL are similar to that of the left limb in our study where it gave a tendon which divided into only three slips.

The presence of an accessory extensor digitorum muscle may be elucidated in relation to the embryological development of the muscles of leg and foot. During the four stages that have been described for muscle pattern ontogenesis, fusion of muscle primordia of different layers forms a muscle [8,9]. Accessory muscle bellies might arise due to persistence of some muscle primordia [10].

The EDL plays an important role in the gait cycle and its size and lower number of tendons might affect the gait cycle [6]. The gait cycle might be altered due to the compression of the tendon of accessory EDL (or EDL) which may restrict the extension of the digits [4]. Thus, the knowledge of such variations helps clinicians to assess the involvement of specific muscles associated with the restricted extension of the toe(s). As the contractures of the toes are thought to be because of EDL, in rare cases these clinical deformities might be associated with the accessory EDL. Knowledge of such variations is important surgically for tendon transfer procedures involving the EDL in case of foot drop [6]. Also, variations determine the feasibility of free tendon grafts, for which the extensor digitorum longus is sometimes used because of its suitable diameter for procedures of reconstruction involving the hand, Achilles tendon and ankle ligaments [7].

The current study reports the presence of an accessory EDL and its asymmetrical variation along with the EDL, which is unique and not reported in previous literatures. Such variations might have clinical implications and might even affect the regular functions of muscles on a whole. Therefore, knowledge of such variations is important in the surgical and orthopaedic fields.
